# *De Novo* Genome and Transcriptome Assembly of the Canadian Beaver (*Castor canadensis*)

**DOI:** 10.1534/g3.116.038208

**Published:** 2017-01-13

**Authors:** Si Lok, Tara A. Paton, Zhuozhi Wang, Gaganjot Kaur, Susan Walker, Ryan K. C. Yuen, Wilson W. L. Sung, Joseph Whitney, Janet A. Buchanan, Brett Trost, Naina Singh, Beverly Apresto, Nan Chen, Matthew Coole, Travis J. Dawson, Karen Ho, Zhizhou Hu, Sanjeev Pullenayegum, Kozue Samler, Arun Shipstone, Fiona Tsoi, Ting Wang, Sergio L. Pereira, Pirooz Rostami, Carol Ann Ryan, Amy Hin Yan Tong, Karen Ng, Yogi Sundaravadanam, Jared T. Simpson, Burton K. Lim, Mark D. Engstrom, Christopher J. Dutton, Kevin C. R. Kerr, Maria Franke, William Rapley, Richard F. Wintle, Stephen W. Scherer

**Affiliations:** * The Centre for Applied Genomics, The Hospital for Sick Children, Toronto, Ontario M5G 0A4, Canada; † Program in Genetics and Genome Biology, The Hospital for Sick Children, Toronto, Ontario M5G 0A4, Canada; ‡ Donnelly Centre for Cellular and Biomolecular Research, University of Toronto, Ontario M5S 3E1, Canada; § Ontario Institute for Cancer Research, MaRS Centre, Toronto, Ontario M5G 0A3, Canada; ** Department of Computer Science, University of Toronto, Ontario M5S 3G4, Canada; †† Department of Natural History, Royal Ontario Museum, Toronto, Ontario M5S 2C6, Canada; ‡‡ Toronto Zoo, Ontario M1B 5K7, Canada; §§ McLaughlin Centre, University of Toronto, Ontario M5S 0A4, Canada; *** Department of Molecular Genetics, Faculty of Medicine, University of Toronto, Ontario M5S 1A8, Canada

**Keywords:** whole-genome sequencing, genome assembly, genome annotation, Canadian beaver, rodent

## Abstract

The Canadian beaver (*Castor canadensis*) is the largest indigenous rodent in North America. We report a draft annotated assembly of the beaver genome, the first for a large rodent and the first mammalian genome assembled directly from uncorrected and moderate coverage (< 30 ×) long reads generated by single-molecule sequencing. The genome size is 2.7 Gb estimated by k-mer analysis. We assembled the beaver genome using the new Canu assembler optimized for noisy reads. The resulting assembly was refined using Pilon supported by short reads (80 ×) and checked for accuracy by congruency against an independent short read assembly. We scaffolded the assembly using the exon–gene models derived from 9805 full-length open reading frames (FL-ORFs) constructed from the beaver leukocyte and muscle transcriptomes. The final assembly comprised 22,515 contigs with an N50 of 278,680 bp and an N50-scaffold of 317,558 bp. Maximum contig and scaffold lengths were 3.3 and 4.2 Mb, respectively, with a combined scaffold length representing 92% of the estimated genome size. The completeness and accuracy of the scaffold assembly was demonstrated by the precise exon placement for 91.1% of the 9805 assembled FL-ORFs and 83.1% of the BUSCO (Benchmarking Universal Single-Copy Orthologs) gene set used to assess the quality of genome assemblies. Well-represented were genes involved in dentition and enamel deposition, defining characteristics of rodents with which the beaver is well-endowed. The study provides insights for genome assembly and an important genomics resource for Castoridae and rodent evolutionary biology.

The Canadian beaver (*Castor canadensis*, Kuhl 1820) is an iconic national symbol. Appreciated for its fur and castoreum, the beaver trade was the economic engine that drove early British and French colonial expansion leading to the founding of Canada. In recognition of this heritage, the beaver appeared on the first Canadian postage stamp issued in 1851 instead of an image of the Queen, which was customary at the time. Indeed, the beaver personifies the Canadian self-identity of being self-reliant, hardworking, and peaceful, but also able to meet challenges in difficult times; traits that lead to its depiction on countless Canadian emblems, corporate seals, and military badges. Once near extinction, the beaver is now a recognized sentinel species for conservation. The beaver is so interwoven in the economic, historical, and societal fabric of Canada, that it received Royal assent in 1975 as the symbol for Canadian sovereignty.

The beaver family, Castoridae, is represented by two extant species with different chromosomal complements (Lavrov and Orlov 1973; Genest *et al.* 1979): *C. canadensis* (Kuhl 1820) in North America and *C. fiber* (Linnaeus 1758) in Eurasia. Little is known of the genomic structures of the *Castor* species. DNA sequencing has produced draft genomes of rodent clades, but these efforts focused on the small rodents. Moreover, genome sequencing mostly relied on short reads. When repetitive sequences are longer than the read length, a unique assembly solution typically cannot be determined among other computationally equivalent alternatives, the number of which grows exponentially with the genome size. Consequently, most assemblies of large genomes generated from short reads alone are highly fragmented. The Pacific Biosciences (PacBio) platform (Pacific BioSciences, Menlo Park, CA) produces reads > 10 kb, with a trailing distribution often exceeding 40 kb, which is sufficient to span many mammalian interspersed repeats. Despite this potential, PacBio reads contain 15–20% errors due to the difficulty of making single-molecule measurements, necessitating genomic coverage of > 55-fold or more for assembly and consensus error correction (Berlin *et al.* 2015; Gordon *et al.* 2016). For large genomes, the cost of obtaining such high coverage is often prohibitive. In a hybrid approach, short reads can be used in a preassembly correction step to reduce the coverage requirement. However, this approach is feasible only for smaller genomes since the computational burden is high (Koren *et al.* 2012).

Here, we present a simplified and less costly approach for useful *de novo* assembly of large genomes, producing the first annotated draft assembly of the Canadian beaver genome to illustrate the feasibility of this approach (Figure 1). We reduced the genomic coverage of noisy long reads to a modest ∼30-fold to reduce cost and sequencing time. We then parameterized the Canu assembler (Koren *et al.* 2016) to produce a primary assembly directly from uncorrected long reads, thereby eliminating the demanding preassembly hybrid correction step using short reads (Koren *et al.* 2012). The final steps involved refining the assembly to remove residual errors, and scaffolding the assembly using exon–gene models derived from *de novo* reconstruction of the beaver leukocyte and muscle transcriptomes.

**Figure 1:**
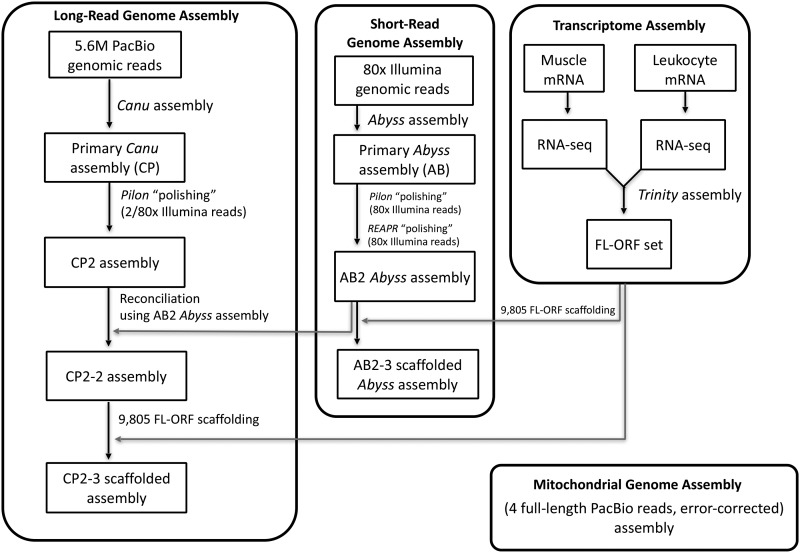
Canadian Beaver Genome Project. Schematic diagram of genome and transcriptome assembly. FL-ORF, full-length open reading frame; PacBio, Pacific Biosciences; RNA-seq, RNA sequencing.

This project was intended to accelerate the transition of genomics into mainstream biology and ultimately precision medicine, both requiring continued improvements in *de novo* assemblies for rare variant detection at cohort or population scales. We release the beaver genome to mark Canada’s sesquicentennial, and hope the initiative will catalyze other exploratory investigations in “cultural genomics;” of which this project was motivated by a nation’s curiosity and the pride in the animal that has most shaped its history.

## Materials and Methods

### DNA and RNA sample collection and isolation

**Figure 2:**
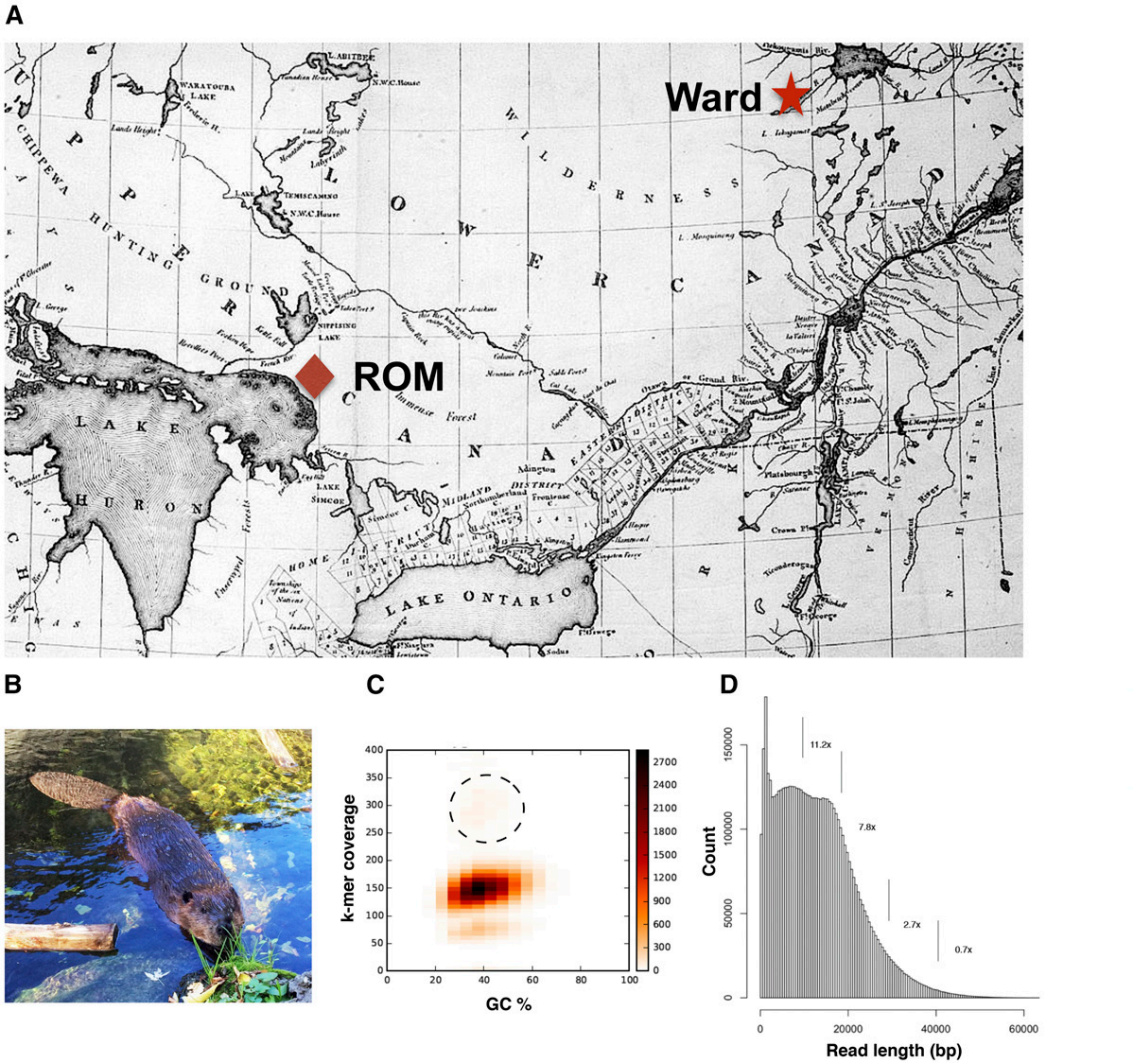
Information on sequenced *C. canadensis*. (A) Map of the Parry Sound region of Upper Canada (Ontario) and the Saguenay-Lac-Saint-Jean region of Lower Canada (Quebec), where the subjects originated. Beaver specimen catalog number ROM 106,880 = red diamond. “Ward” beaver, the Toronto Zoo = red star (Map: Fensett circa 1812, Wikimedia Foundation/public domain). (B) Image of “Ward” at the Toronto Zoo (Photo: courtesy of the Toronto Zoo). (C) Size estimation of beaver genome by k-mer analysis. The circled region denotes faint signals from repetitive sequences. (D) PacBio read length distribution from 80 flow cells; 5.6 million reads representing ∼30-fold coverage of beaver genome. The histogram is further divided into fold-coverage of the genome in the 10 kb read length intervals as indicated. PacBio, Pacific Biosciences; ROM, Royal Ontario Museum.

Blood from a 10-yr-old male beaver (named “Ward”), residing at the Toronto Zoo, was collected by veterinary personnel in accordance with approved institutional procedures and protocols. Ward is a captive-bred Canadian beaver born at Zoo Sauvage de St. Felicien (Quebec) from parents collected from the wild in the Saguenay-Lac-Saint-Jean region of Quebec (Figure 2A). Blood was collected using the BD Vacutainer Safety-Lok Blood Collection Set (Becton Dickinson, Franklin Lake, NJ), with 4 ml blood for DNA isolation in an EDTA Blood Vacutainer and 2.5 ml for RNA isolation in PAXgene RNA Tubes. Samples were transported at room temperature and processed within 24 hr. Beaver muscle tissue for transcriptome analysis was provided by the Royal Ontario Museum (ROM) from frozen archival tissue (*C. canadensis*, catalog number ROM 106880, Maple Island, Dunchurch, Parry Sound District, Ontario, Canada) in accordance with institutional guidelines.

We purified DNA for genome sequencing from fresh blood using the Puregene Chemistry on an Autopure LS DNA Extractor (QIAGEN, Hilden, Germany). Genomic DNA was quantified by fluorometry using Qubit DNA HS Assay (ThermoFisher, Waltham, MA); assessment of its physical integrity showed a central main peak of 55 kb mean length on an Agilent TapeStation (Agilent, Santa Clara, CA). RNA for transcriptome sequencing was purified from blood leukocytes using a QIAsymphony PAXgene Blood RNA Kit (QIAGEN). Muscle RNA for transcriptome sequencing was isolated from 60 mg of −80° frozen tissue using the PerfectPure RNA Tissue Kit (5Prime, Gaithersburg, MD). Lysis solution and TCEP provided were added directly to frozen tissue, which we homogenized using an Omi TH Homogenizer (Omni International, Kennesaw, GA) and a disposable probe. Homogenate was precleared and loaded onto RNA purification columns as per kit protocol. We quantified RNA integrity on an Agilent Bioanalyzer RNA Nano chip (Agilent), with reporting integrity numbers (RIN) of 7.7 and 8.1 for RNA isolated from fresh blood leukocyte and frozen muscle, respectively. RNA concentration was determined by fluorometry using a Qubit RNA HS Assay (ThermoFisher).

### cDNA library construction, sequencing, and preprocessing

RNA-seq analysis was performed at The Centre for Applied Genomics (TCAG) in Toronto. Samples were depleted of ribosomal RNA (rRNA) using the NEBNExt Ultra Directional RNA Library Prep Kit (New England BioLabs, Beverly, MA), and were sequenced on the Illumina HiSeq 2500 platform using V4 Chemistry (Illumina, San Diego, CA) to generate paired-end reads of 126 bases from 300–400 base inserts. In brief, Illumina-compatible adaptors were ligated to the cDNA and amplified for 12 cycles, during which different sample-barcoded primers were incorporated to enable multiplexed sequencing. The amplified cDNA was quantified for loading onto the sequencing flow cell using a KAPA Library Quantification Kit (Roche Diagnostics, Laval, Quebec, Canada). The resulting sequencing runs generated 120 and 166 million paired-end reads from the leukocyte and muscle libraries, respectively. Reads were trimmed of adaptor and low-quality sequences using Trimmomatic v0.32 (Bolger *et al.* 2014), discarding reads shorter than 36 bases. We then employed QuorUM v1.0.0 (Marcais *et al.* 2015) and a k-mer size of 24 to correct the trimmed sequence reads.

### De novo transcriptome assembly and annotation using reference species

We assembled the beaver muscle and blood leukocyte transcriptomes from error-corrected strand-specific reads using Trinity (Grabherr *et al.* 2011), filtered through TransDecoder v2.1.1 to identify potential coding sequences. Assembled full- or partial-length candidate coding sequences, referred to as Trinity components, were compared to known protein sequences from the *Mus musculus* reference genome version GRCm38 using BLASTp. If a significant BLAST hit was not found in mouse, we extended the search to annotated reference proteins of other species in the order indicated: *Rattus norvegicus* (Norwegian brown rat), *Dipodomys ordii* (Ord’s kangaroo rat), *Microtus ochrogaster* (prairie vole), *Chinchilla lanigera* (long-tailed chinchilla), *Peromyscus maniculatus* (North American deer mouse), *Marmota marmota* (alpine marmot), and *Ictidomys tridecemlineatus* (13-lined ground squirrel). When necessary, we included two non-rodent reference species in this process: *Homo sapiens* (human) and *Pan troglodytes* (common chimpanzee).

We matched potential coding regions to the best BLASTp hit in the mouse reference protein set using an *e*-value cutoff of 1e−10. We merged Trinity components to create longer ORFs if all the following conditions were met: (1) if the components identified the same reference protein as the best (or near best) BLAST hit; (2) if the BLAST hits to the reference protein by each Trinity component differed by ≤ 15%; (3) if the Trinity components shared at least 15 overlapping nucleotide bases; and (4) if the newly created reading frame from the merging maintains similarity to the reference protein. Cases were manually curated when some but not all of the above conditions were met.

The BLAST match of the Trinity component would sometimes not extend to the N- or C-terminus of the reference protein due to reduced sequence conservation or variable numbers of bases at the start or end, which are often found in orthologous proteins. When the BLAST match region did not include the reference protein’s start codon, we assigned a provisional initiation codon if the Trinity component had an in-frame ATG ≤ 10 amino acids (30 nucleotides) upstream of the BLAST match region of the presumptive protein. We used a similar rationale to assign the provisional stop codon. ORFs were considered FL-ORFs when the Trinity components contained both start and stop codons and encoded a contiguous polypeptide with substantial similarity to a reference protein along its length. Trinity components that partially covered the reference sequence or were missing bases at the start, end, or middle were denoted as partial-length ORFs (PL-ORFs). For genes encoding multiple protein isoforms (principally due to alternative splicing), we selected the FL-ORF corresponding to the longest isoform for each gene, presumably the ORF comprising the most coding exons, to represent the beaver locus. Other isoforms encoded by the same gene, if constructed or identified, were marked as secondary and kept in a separate bin.

Through the steps outlined above, gene symbols of the matching reference protein sequences were provisionally assigned to the assembled FL- or PL-ORFs. Further, we compared the *M. musculus* exon–gene model of the reference proteins to these ORFs to demarcate potential exon boundaries for genome annotation and scaffolding. During this process, we accommodated insertions and deletions in the BLAST match with the predicted exon boundaries adjusted accordingly.

### Genome sequencing

#### PacBio SMRT DNA sequencing:

We assembled the beaver genome using a strategy whereby a primary assembly was generated using moderate-coverage, uncorrected long reads from the PacBio RSII SMRT sequencer. Long read sequencing using the PacBio RSII SMRT platform was performed at the Ontario Institute for Cancer Research (OICR) in Toronto. PacBio SMRTbell libraries were constructed from purified genomic DNA from fresh beaver blood leukocytes. The DNA shearing step was omitted since pulsed-field gel analysis showed that genomic DNA isolated from Ward already had a broad size distribution with a peak mode length of ∼40 kb, similar to the recommended size for library construction. Libraries were prepared from 15 μg of genomic DNA using the SMRTbell Template Prep Kit 1.0, according to the manufacturer’s instructions for 20 kb insert templates. SMRTbell libraries were size-selected using the BluePippin System (Sage Science, Beverly, MA) with a lower cut-off of 15 kb. We performed whole-genome SMRT sequencing using 80 flow cells, running the current P6/C4 chemistry with a 6 hr movie-time, and used PacBio’s P-Filter Module protocol to process raw data to remove library adaptors and redundant or low-quality sequence reads. Each of the 80 flow cells produced an average of 1 Gb of filtered PacBio reads with average read length of ∼13–14 kb, and together represented ∼30-fold coverage of the beaver genome.

#### Illumina paired-end DNA sequencing:

We performed whole-genome, short read sequencing at TCAG on the Illumina HiSeq X platform. Purified genomic DNA from fresh beaver blood leukocytes was quantified by Qubit DNA HS Assay (ThermoFisher). Genomic libraries were constructed using the Illumina Nano DNA Library Preparation Kit from DNA sheared using a Covaris LE220 Focused Ultrasonicator (Covaris, Woburn, MA) to an average length between 300 and 400 bases. We used 100 ng of sheared DNA for library preparation, following Illumina’s recommendation, and six amplification cycles. Libraries were quantified for sample loading using the KAPA Library Quantification Kit (Roche Diagnostics). Four lanes of DNA sequences (150 base paired-end reads of a 300–400 base insert) were produced, representing an estimated 160-fold coverage of the genome.

### Size estimation of the beaver genome

The size of the beaver genome was estimated by tabulating the k-mer frequencies of 160 million Illumina reads using preQC (Simpson 2014). Prior to analysis, Illumina paired-end reads (150 base paired-end reads of 300–400 base insert size) were corrected using QuorUM (Marcais *et al.* 2015) at a k-mer size of 24.

### De novo genome assembly

The beaver genome was assembled using a strategy whereby moderate genome coverage long PacBio reads were assembled directly into a primary draft genome without prior error correction of the individual reads. Figure 1 shows a schematic diagram of the assembly workflow. We used the new Canu assembler v1.2 (Koren *et al.* 2016) with parameters optimized for noisy reads to assemble 5.6 million trimmed SMRT reads of 14.4 kb average read length. These sequences represent 30-fold estimated coverage of the beaver genome. Before assembly, we removed 91 reads surmised to have originated from mitochondria, based on similarity to a recently reported beaver mitochondrial genome (Horn *et al.* 2011) and the absence of presumptive flanking nuclear sequences. Removal of mitochondrial sequences was done to prevent the possibility of inducing misassembly with the mitochondrial pseudogene loci reported to exist in the nuclear genomes of rodents (Richly and Leister 2004). Assembly of uncorrected PacBio reads was performed using Canu’s parameterized “high-sensitivity mode” (corMhapSensitivity = high; corMinCoverage = 0; errorRate = 0.035; minOverlapLength = 499) to yield the primary assembly, referred to as “CP.”

We subjected the CP assembly to two separate refinement or polishing steps. The first step comprised two consecutive rounds using the program Pilon (Walker *et al.* 2014) in the “fix-all” mode, in which each round was supported by an independent bolus of 80-fold (estimated) genome coverage of Illumina paired-end reads (2 × 150 base, 300–400 base insert). Pilon was originally developed for microbial genomes, but it could be scaled to process larger genomes with the addition of more memory. We followed the developer’s recommendation of allocating 1 Gb memory to the JVM (Java Virtual Machine) per Mb of assembled contigs to be polished (github.com/broadinstitute/pilon/wiki/Requirements-&-Usage). Illumina reads were first mapped onto the assembled beaver contigs using BWA (Li and Durbin 2009) and the mapped contigs were divided into ∼100 Mb batches. Each batch was assigned 100 Gb of memory on a 40-core Dell machine, with run time of ∼2 hr using Pilon v1.16. After the second round of Pilon, the assembly was referred to as “CP2.”

The second polishing step used a high-quality short read assembly to test for discordance and provided a second and independent check for local misassembly at the Canu assembly junctions. The short read assembly used for this purpose was constructed from 80-fold genome coverage of Illumina short reads using the program Abyss (Simpson *et al.* 2009) and a k-mer length of 97. Before use, the Abyss assembly (AB) was polished using Pilon in the “fix-all” mode supported by 80-fold Illumina paired-end reads. The resulting polished assembly, “AB1,” was subjected to aggressive trimming of potential misassembled regions using the program REAPR (Hunt *et al.* 2013) to ensure the highest quality Abyss assembly possible. The resulting assembly after the REAPR step is referred to as “AB2.”

For the final polishing step of the CP2 assembly, we first map Canu-corrected and trimmed PacBio reads onto CP2 using BLASR, which was developed for mapping of noisy long reads from single-molecule sequencing (Chaisson and Tesler 2012). We then mapped AB2 contigs onto CP2 using BLASTn (Atschul *et al.* 1990). AB2 contigs with termini within a 500 bp window that did not map significantly to CP2 were deemed to be potential sites of misassembly in either the Abyss AB2 assembly or (more likely) in the PacBio CP2 assembly. To err on the side of accuracy, any discordant regions thus identified on CP2 that were not supported by one or more mapped PacBio reads were broken at the discordant regions. The final assembly after this process is referred to as “CP2-2.”

### Scaffolding and validating genomic assembly using the transcriptome

To scaffold the resulting long read genomic assembly, CP2-2, we used the predicted exon–gene models of 9805 *de novo* assembled and annotated beaver FL-ORFs, and 4045 PL-ORFs with ≥ 90% coverage of a rodent reference protein. We divided sequences from each FL- or PL-ORF into exons according to the predicted boundaries, and saved each in an exon FASTA file. We used BLASTn to align each predicted exon sequence onto CP2-2 with > 98% sequence identity over the entire length of the exon (with 10 bp flexibility at the predicted exon boundaries to account for possible split codons and minor species differences in splice site selection). The resulting BLASTn output was provided to a custom written scaffolder program, which is available upon request. The scaffolder takes ORFs one at a time and sorts to derive the maximum possible number of exons in as few contigs as possible, with the best identity score. It then sorts the ORFs initially into three broad categories. The first category comprised ORFs whose entire complement of predicted exons is present in the correct order and orientation on a single assembled contig, thereby providing an independent assessment of assembly accuracy for that contig, based on the congruity of the exon–gene model. The second category comprised FL- or PL-ORFs whose complement of mapped exons was congruent to the exon–gene model, but whose exon complement spanned two or more assembled contigs, thereby enabling these ORFs to scaffold multiple contigs. The third category comprised ORFs that were missing one or more predicted exons in the CP2-2 assembly, or had mapped exons incongruent with the exon–gene model. Accordingly, ORFs in the third category might reflect potential gaps or errors in the CP2-2 assembly, misassembly of the actual ORF, or the use of an incorrect exon–gene model for the ORF, all of which required further inspection. Missing exons in category 2, whose predicted size was < 17 bp in length, were noted but were spared, since they are below the sensitivity threshold of the BLAST search. We searched the secondary assemblies (*e.g.*, unused PacBio sequences and Illumina Abyss assembly) for missing exons and, when found, incorporated them into the assembly as a component of a scaffold. In rare cases where the exon–gene model indicated that an exon might be in the reverse orientation or in the incorrect order in the assembly, we flagged these regions for manual inspection. We undertook manual curation only if they could be supported by independent sequence reads; otherwise, these regions of potential misassemblies were simply flagged for future resolution. The resulting scaffolded assembly is referred to as “CP2-3,” in which a string of 100 “N”s denotes the scaffold joint.

### Data availability

All sequences and data contributing to the assemblies are already submitted, or are in the process of submission, to GenBank and other public archives and repositories. This study has been assigned BioProject number PRJNA359140 to facilitate data dissemination. 

## Results and Discussion

Figure 1 presents the schematics of the assembly workflow as described in the *Materials and Methods*. For genome sequencing, we selected a male beaver named Ward, a descendent from the Saguenay-Lac-Saint-Jean population of Quebec. This region had a rich history in the fur trade and was the jurisdiction of two fur trading rivals, the North West Company and later the Hudson’s Bay Company. Beaver pelt was a “territorial currency” in the region until the early 20th century (Gettler 2013). Figure 2A shows a historical map of the region in Quebec (Lower Canada) where subject Ward originated, and the Parry Sound region of Ontario (Upper Canada), where the ROM sample was collected. Figure 2B shows beaver Ward, now residing at the Toronto Zoo with his partner, June.

### De novo assembly of C. canadensis muscle and leukocyte transcriptome

**Figure 3:**
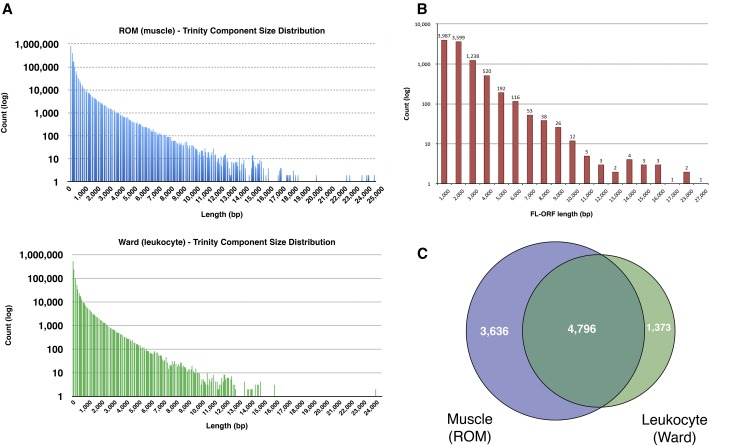
*C. canadensis* transcriptome analysis. (A) Size distribution of the assembled Trinity components from muscle and leukocyte RNA-seq reads. (B) Length distribution of assembled FL-ORFs from leukocyte and muscle transcriptomes. (C) Distribution of FL-ORFs in leukocyte and muscle. FL-ORF, full-length open reading frame; RNA-seq, RNA sequencing; ROM, Royal Ontario Museum.

Transcriptome sequencing provided the first glimpse of the beaver genome. *De novo* transcriptome assembly was performed on RNA of two beaver tissues using the program Trinity (Grabherr *et al.* 2011) to generate full-length ORFs for quality assessment, scaffolding, and annotation of the genomic assembly. Figure 3A presents the size distribution of over 200,000 Trinity components assembled independently from 160 to 120 million 126 base paired-end reads from random-primed rRNA-depleted RNA from frozen archival muscle tissue (ROM sample), and from fresh beaver blood leukocytes (Ward), respectively. Figure 3B presents the size distribution of 9805 high-confidence FL-ORFs identified from BLASTp analysis of assembled Trinity components against reference panels of annotated rodent proteins, as described in the *Materials and Methods*. As these FL-ORFs typically encode polypeptides having > 90% identity to the rodent reference proteins, we are confident about provisional ortholog and locus assignments to these FL-ORFs as the first step in annotating the beaver genome. Based on these assignments, 2113 of the 3013 genes (70%) in the BUSCO gene set (Simao *et al.* 2015) are represented in our collection of beaver FL-ORFs. In addition, 4045 PL-ORFs were assembled to which provisional locus assignments could also be made. Typically, these PL-ORFs are missing only a small portion of the coding region corresponding to the extreme N-terminus of the reference protein, reflecting the inherent underrepresentation of that region of mRNA during library construction. Although these PL-ORFs were not full-length relative to the predicted coding sequences, they nevertheless contributed to the annotation and the scaffolding of the genome assembly.

Figure 3C presents the expression distribution of the FL-ORFs in blood leukocyte and muscle tissue. Within the sensitivity specified by the RNA-seq read depth, 4796 loci are commonly expressed in the two tissues, expression of 3636 loci are detectable only in muscle, and expression of 1373 loci are detectable only in leukocytes. Although the analysis of two tissues does not represent a comprehensive transcriptional profile of the beaver, *de novo* assembly of 9805 FL-ORFs and 4045 PL-ORFs represents nearly half the gene complement in a typical mammalian genome, thereby providing a useful resource for phylogenetic studies and for validating and scaffolding the genome assembly. A complete list of the assembled ORFs, their ortholog assignments, and sequences can be accessed in Supplemental Material, Table S1, available online.

### Beaver mitochondrion assembly

Mitochondrial reads were present at very low frequency in the PacBio sequence data since the library was constructed without a DNA shearing step, the absence of which biased the representation of circular mitochondrial DNA. Nevertheless, from > 5 million PacBio reads, we identified four reads longer than 16 kb, which essentially spanned the complete mitochondrial genome. These reads were similar along their entire lengths to a reported complete beaver mitochondrial genome sequence derived from amplicon pyrosequencing (Horn *et al.* 2011), and contained no extraneous presumptive genomic sequences indicative of the reads originating from mitochondrial pseudogenes in the nuclear genome. We believed these reads were created from the chance linearization of the circular mitochondrial genome during DNA purification and were incorporated into the PacBio library. Consistent with having originated from the beaver mitochondria, these reads could be assembled into a circular permuted molecule of 16,767 bp with the expected hallmarks of a mitochondrial genome. The resulting uncorrected mitochondrial assembly from the PacBio reads is referred to as “mito-0.”

Consensus base-calling with aligned Illumina short reads is typically used to correct sequence errors inherent to the PacBio platform. Unlike the PacBio library, the Illumina short read library was constructed from sheared leukocyte genomic DNA in which circular mitochondrial genomic DNAs are fragmented and are proportionally represented in the short read library. For error correction, we aligned Illumina genomic short reads from Ward beaver leukocytes across the mito-0 draft assembly at > 10,000 × depth using Bowtie (Langmead *et al.* 2009). We then used Pilon v1.16 to derive a complete consensus-corrected mitochondrial sequence, referred to as “mito-1” (GenBank accession number KY311838).

**Figure 4:**
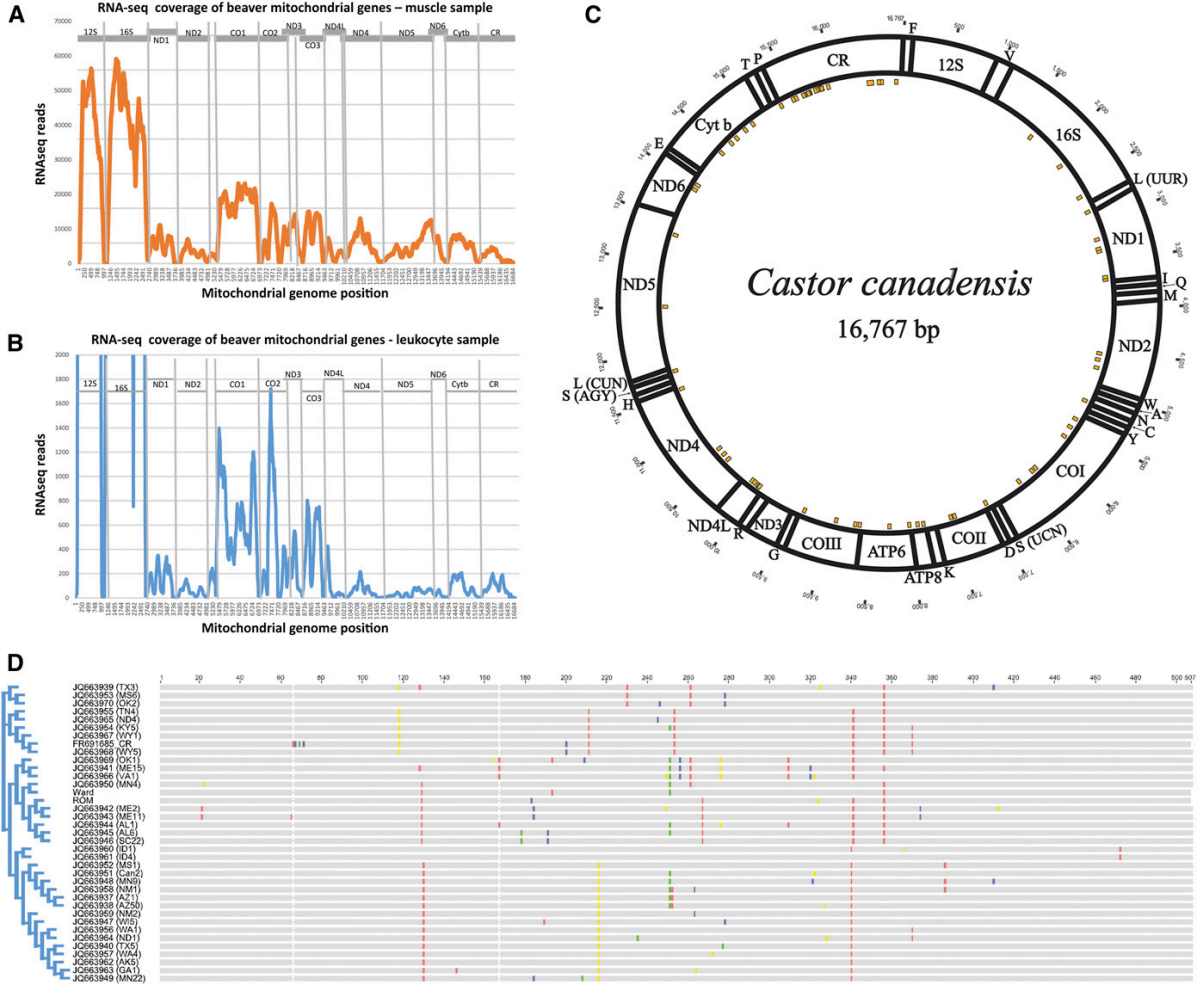
Analysis of *C. canadensis* mitochondria. (A) Tiling depth of mitochondrial RNA sequencing (RNA-seq) reads from archival muscle tissue. (B) Tiling depth of mitochondrial RNA-seq reads from leukocytes. (C) Diagrammatic depiction of *C. canadensis* mitochondrial genome. ND, NADH dehydrogenase; CO, Cytochrome c oxidase; ATP, ATPase; Cyt b, Cytochrome b; and CR, Control Region (including D-loop). Transfer RNA (tRNA) genes are denoted by the one-letter amino acid code. Variants between the leukocyte assembly and the Horn *et al.* (2011) assembly are shown with gold rectangles (see also Table 4). (D) Alignment of 500 bp of D-loop sequence downloaded from GenBank with *C. canadensis* samples from this study, with neighbor-joining tree. Branch lengths have been formatted (proportional transformation) for space considerations. Samples Ward (KY311838) and ROM (Royal Ontario Museum) (KY321562) are as indicated. Horn sample, *C. canadensis* harvested in Finland (Horn *et al.* 2011) (FR691685). Pelz Serrano samples (Pelz Serrano 2011) (JQ663937-70): AK, Alaska; AL, Alabama; Can, Alberta; GA, Georgia; ID, Idaho; KY, Kentucky; ND, North Dakota; NM, New Mexico; ME, Maine; MN, Minnesota; MS, Mississippi; OK, Oklahoma; SC, South Carolina; TN, Tennessee; TX, Texas; VA, Virginia; WA, Washington; WI, Wisconsin; and WY, Wyoming.

Rodent genomes can contain several hundred copies of mitochondrial pseudogene fragments, termed “NUMTs” (nuclear copies of mtDNA) (for a review, see Richly and Leister 2004). NUMTs could accumulate mutations, and genomic reads originating from such NUMTs might interfere with consensus error correction of the mitochondrial genome assembly, mito-0. Since NUMTs are believed to be transcriptionally inactive, we compared the result of consensus correction of mito-0 using RNA-seq reads with the result of using genomic reads from the same beaver. Ward leukocyte RNA-seq reads covered 96% of the mito-0 assembly (depth > 5×) (Figure 4A). When tiled RNA-seq reads from this region were used for the consensus error correction, we found that the corrected sequence was identical to the corresponding region of the mito-1 assembly that was corrected using genomic DNA reads. This result indicates that the level of NUMT sequences in the genomic reads do not materially affect consensus error correction of mito-0.

For the ROM beaver specimen, for which we did not produce genomic reads, the mitochondrial genome was assembled from RNA-seq reads. For this individual, 1,496,670 muscle-derived RNA-seq reads that could be mapped onto the mito-0 assembly achieved at least 5 × coverage across 99.5% of the mito-0 template. The majority of the remaining portion of mito-0 has at least one RNA-seq read (Figure 4B). As expected, the highest RNA-seq mapped depth spanned the major coding sequences of the mitochondrial genome, with the lowest depth observed across the tRNA genes and the noncoding control region. From nearly 1.5 million tiled RNA-seq reads, we constructed a consensus muscle mitochondrial genome, “mito-2,” for the ROM beaver (GenBank accession number KY321562) using Geneious r7.1.9 (http://www.geneious.com; Kearse *et al.* 2012).

Mito-1 from Ward beaver is similar in size and structure to a mitochondrial genome previously reported for *C. canadensis* generated by amplicon pyrosequencing (Horn *et al.* 2011). We found 68 variant positions between the two sequences (Table 4 and also indicated in Figure 4C). Sixty of these are single nucleotide variants (SNV) (56 transitions and four transversions), six are small insertion/deletions (indels), and two are more complex substitutions. Based on the mito-1 assembly, which was anchored by long PacBio reads, we determined the exact length of the tandem repeat embedded in the control region (start position 16,169) to be 320 bases (32 bases repeated 10 times). Since the Horn assembly (Horn *et al.* 2011) was derived from pyrosequencing of amplicon-derived segments, where a number of positions were curated to maintain the translation frame, it is not clear whether the differences observed between the two genomes are real or are due to residual uncorrected pyrosequencing artifacts.

We observed 34 SNVs between the mitochondrial genomes of Ward (mito-1) and the ROM specimen (mito-2) (Table 5). To compare our specimens with those of other Canadian beavers, we aligned 504 bp of the control region of our samples to 35 other *C. canadensis* control region sequences downloaded from GenBank (JQ663937-70; Pelz Serrano 2011 and FR691684; Horn *et al.* 2011) using ClustalW implemented in Geneious r7.1.9. Unrooted trees were constructed under neighbor joining (NJ) or maximum likelihood (ML) methods using PAUP* v4.0a150 (Swofford 2002), with the HKY substitution model estimated using ModelTest implemented in PAUP*. Although deeper nodes of the tree were unresolved, both ML and NJ phylogenetic trees (NJ tree and alignment in Figure 4D) showed that the control regions in our samples are most similar to haplotypes from beavers collected in the northeastern United States and assigned to the Atlantic Slope Drainage System (Pelz Serrano 2011). Unfortunately, beaver populations in Eastern Canada were not investigated in the Pelz Serrano study.

### Genome assembly, validation, and annotation

To our knowledge, the present work represents the first large complex mammalian genome assembled directly from moderate genomic coverage (∼30 ×) of uncorrected long reads generated by single-molecule sequencing. As proof of concept, we used a new version of Canu (v1.2) (Koren *et al.* 2016), a fork of the Celera Assembler specifically designed for hierarchical assembly of low-coverage noisy reads. Previously for large genomes, at least 54-fold coverage was reported for hierarchical assembly using long uncorrected reads to achieve accurate consensus error correction prior to assembly (Berlin *et al.* 2015; Gordon *et al.* 2016). The ability of Canu to assemble lower coverage uncorrected reads directly can lower the cost for *de novo* single-molecule sequencing by at least a factor of two, while bypassing the need for a computationally-intensive, preassembly error correction step (Koren *et al.* 2012). As a consequence of the simplified workflow for *de novo* assembly presented in this study, further development of this approach has the potential for cost-effective scaling to study large cohorts, including population or disease-focused human samples.

### Estimation of beaver genome size and level of heterozygosity

We estimated the beaver genome size using preQC (Simpson 2014) based on k-mer analysis of 160 million Illumina short reads. The scatterplot produced by preQC showed two major components, which is indicative of a genome with an apparent high level of heterozygosity (Figure 2C). A third minor component, barely visible in the plot (demarcated by dashes for clarity in Figure 2C), stemmed from repeats in the genome. The tabulation of k-mer frequencies estimated a haploid genome size of ∼2.7 Gb.

The high level of genomic heterozygosity suggested by k-mer analysis is supported by the direct tabulation of heterozygous positions observed during genome assembly (see below); of the 2.486 Gb of combined contigs in our final beaver genome assembly, the Pilon program tabulated 4,433,832 potential single nucleotide heterozygous positions (0.18% of the assembled sequences) during the assembly-polishing step using Illumina short reads. Based on these metrics, the beaver genome contains threefold more heterozygosity compared with the human genome, where heterozygous positions comprised 0.06% of the genome (Yuen *et al.* 2015).

### Beaver genome assembly

Figure 2D shows the combined size distribution of adaptor-trimmed PacBio reads generated from sample Ward from 80 flow cells. The data set comprised 5,646,602 reads (maximum length 72,608 bases; minimum length 2500 bases; median length 13,247 bases; and mean length 14,403 bases). The data set has a significant trailing distribution of reads exceeding 40 kb. This fraction is inherently the most valuable contributory factor for a superior assembly due to its ability to span long repeats, but could only be accumulated at the expense of running multiple flow cells. Our study has combined PacBio reads totaling 80 Gb, representing ∼30-fold coverage of an estimated genome size of 2.7 Gb. Figure 2D shows that ∼0.7 × coverage of the beaver genome is represented by PacBio reads longer than 40 kb. As recommended by the developers of the program, all 5.6 million PacBio reads were assembled in a single bolus using Canu’s high-sensitivity mode. Assembly using Canu v1.2 involved three sequential operations: (1) self-error correction using input reads; (2) trimming of spurious sequences; and (3) assembly using adaptive k-mer weighting and repeat separation. Table 1, Table 2, and Table 3 show the summary assembly statistics of 5.6 million PacBio reads as they transit through the three operational stages of Canu v1.2, the subsequent polishing steps, and final scaffolding of the assembly using the transcriptome.

**Table 1:** Assembly and scaffolding statistics for the *C. canadensis* genome: Canu v1.2 – PacBio preassembly read usage

	PacBio Reads (Gb)	Gb Lost (%)
Input PacBio reads	5,646,491 (80)	
Reads after error correction step	5,568,093 (78)	2.5
Reads after trimming step	4,546,175 (48)	40.0

PacBio, Pacific Biosciences.

**Table 2:** Assembly and scaffolding statistics for *C. canadensis* genome: Canu v1.2 assembly metrics from 4.546 M input PacBio reads (48 Gb)

	Version Name	Number of Contigs	N50 (bp)	Total Bases (Gb)	Max Contig Length (Mb)
Primary Canu assembly	CP	11,982	538,502	2.484	4.666
First Pilon polishing	CP1	11,982	539,399	2.488	4.678
Second Pilon polishing	CP2	11,981	539,125	2.487	4.677
Assembly reconciled with Abyss assembly	CP2-2	22,515	278,680	2.487	3.330
Assembly scaffolded with FL- and PL-ORFs	CP2-3	21,170	317,558	2.518	4.235
Unused PacBio reads (Singletons)	Unused	188,572	14,524	1.803	0.074

PacBio, Pacific Biosciences; ORFs, open reading frames.

**Table 3:** Assembly and scaffolding statistics for *C. canadensis* genome: Abyss assembly metrics from 80 M input Illumina reads (24 Gb) (PE 150 bp, 300-400 bp Insert)

Abyss Assembly	Version Name	Number of Contigs	N50 (bp)	Total Base (Gb)	Max Contig Length (Mb)
Primary assembly	AB	312,881	17,834	2.431	0.206
Pilon correction	AB1	312,881	17,834	2.431	0.261
REAPR correction	AB2	316,195	17,517	2.431	0.261

As shown in Table 1 and Table 2, 5.6 million input PacBio reads gave rise to a Pilon polished assembly, referred to as “CP2,” comprising 11,981 assembled contigs with an N50 of 539,125 bp and a maximum contig length of 4.677 Mb. The CP2 assembly had a combined contig length of 2.487 Gb, representing ∼92% of the estimated 2.7 Gb size of the beaver genome. Showing the advantage of using long reads over short reads for assembly, the CP2 assembly metrics compared favorably with an optimized high-quality short read assembly, generated using Abyss from 80-fold genome coverage of the same genomic DNA (Table 3). Although the majority of the Abyss contigs were contained within the CP2 assembly and had similar overall combined contig length, the Abyss assembly (AB2) was expectedly much more fragmented, comprising over 316,195 contigs with an N50 of only 17,517 bp.

Table 1 shows that the Canu program’s self-error correction and trimming steps eliminated ∼40% of the input PacBio sequences before the assembly stage of the program. This loss might be due to poor sequence quality, the collapsing of internally contained reads, or other related causes. With this loss of reads, the beaver genome was effectively assembled from < 16-fold genomic coverage of usable PacBio sequences. The Canu developers have indicated that up to a third of input sequences might be discarded or consumed in the self-correction and trimming steps, prior to the actual assembly. Our higher rate of loss at these stages might reflect a lower quality of input PacBio reads compared with the norm, although our data met or exceeded the PacBio performance metrics for each flow cell. Other investigators also reported a significant loss of PacBio sequences during error correction. In the case of the parrot genome, 30% of the PacBio sequences were lost (or were not correctable and were discarded) during the preassembly error-correction step using Illumina reads (Koren *et al.* 2012).

A companion output of the Canu assembler was a sizeable unassembled fraction termed “Unused PacBio reads” (Table 2). This unused fraction was comprised of singleton reads and “pseudo-singleton” reads that did not participate in the primary assembly. Pseudo-singleton reads amalgamated other unused reads in their entirety, but without extending the length of the original singleton reads. PacBio reads have random errors in the 15–20% range (Chaisson and Tesler 2012); however, this rate is the average across millions of individual single-molecule sequencing reactions. Some single-molecule sequencing reactions within the flow cell might have error profiles deviating significantly from the mean, and the unassembled fraction in our study might represent reads whose error rate exceeded the operational capacity of Canu to handle noisy data, and were thus not used in the assembly. We have not studied the properties of the CP-unassembled fraction in depth, but together they represented 1.8 Gb of sequence (∼4% of the input DNA).

**Figure 5:**
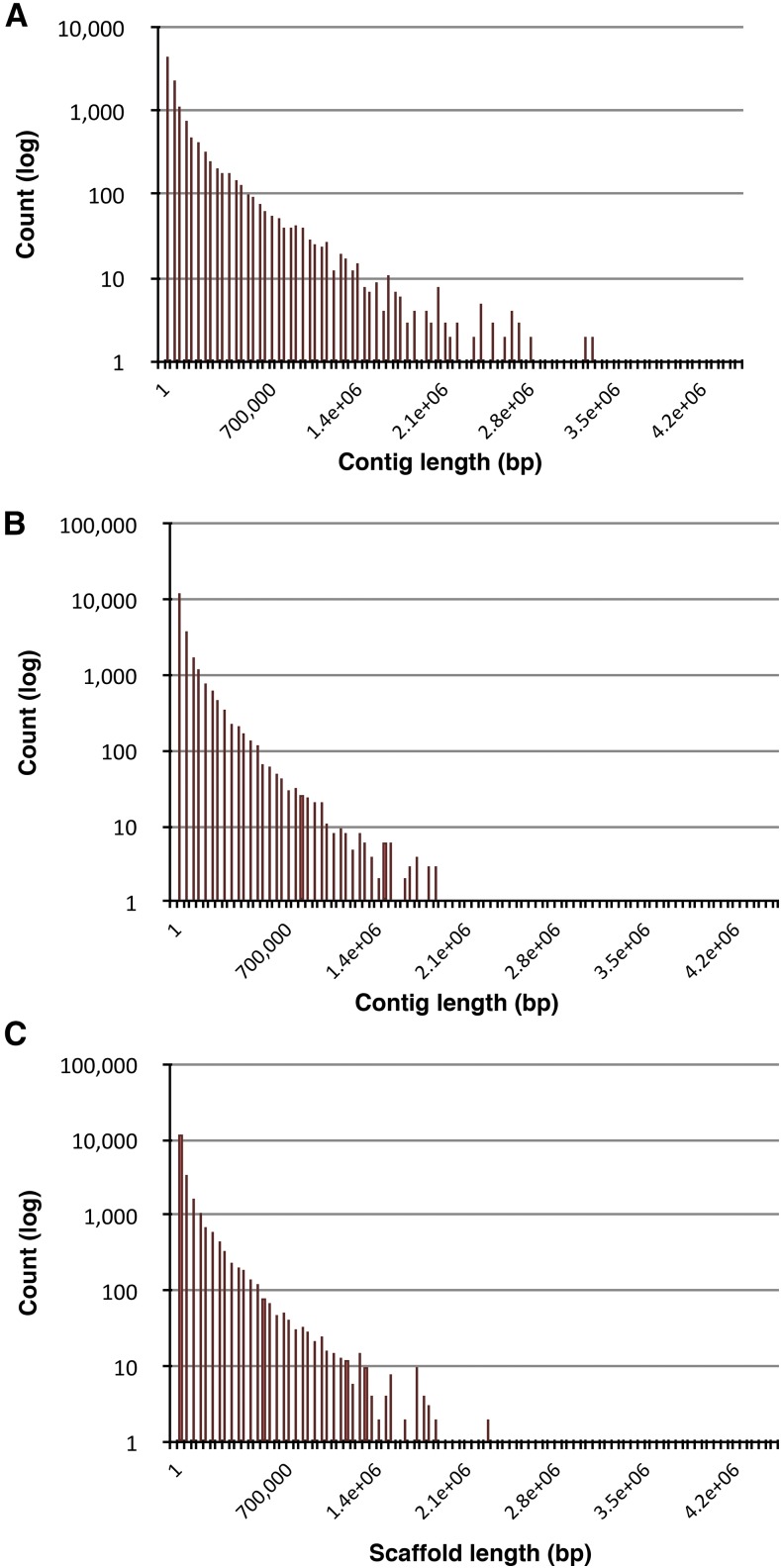
Contig length distributions. (A) CP2 assembly: Canu assembly and Pilon polishing. (B) CP2-2 assembly: breakage of regions of discordance with Abyss assembly (AB2). (C) CP2-3 assembly: scaffolding with FL- and PL-ORFs, and the BUSCO gene set. BUSCO, Benchmarking Universal Single-Copy Orthologs; FL-ORF, full-length open reading frame; PL-ORF, partial-length open reading frame.

The program Pilon was developed to polish PacBio genome assemblies using Illumina short reads (Walker *et al.* 2014). We applied Pilon twice, each time using a different bolus of Illumina reads, to achieve a greater polishing of our primary Canu assembly. As indicated in Table 2, the effects of Pilon on the assembly metrics were subtle, with minimal changes in contig number or the assembly N50, after both one round (CP1) and after two rounds (CP2) of Pilon. To achieve a higher quality genome assembly, we subjected the Pilon-polished CP2 assembly to an additional polishing step, whereby we compared the CP2 assembly against a high-quality, Abyss-derived short read assembly to identify incongruities for remediation. The short read assembly for this purpose (AB2) was assembled from 80-fold genome coverage of Illumina paired-end reads (300–400 base insert) using the Abyss assembler under optimized conditions as described in *Materials and Methods* (Table 3). We mapped AB2 contigs assembled using Abyss, and PacBio reads (trimmed by Canu), onto the CP2 assembly. As described in the *Materials and Methods*, any regions within CP2 that were discordant with the termini of the Abyss contigs that were not supported by one or more mapped PacBio reads were broken at the discordant region on the CP2 assembly. As shown in Table 2, the resulting assembly through this process, referred to as “CP2-2,” showed a marked increase in contig number (from 11,981 to 22,515) and a nearly twofold reduction in the N50 metric (from 539,125 to 278,680 bp). Figure 5, A and B show the relative size distribution of assembled contig lengths before (CP2) and after (CP2-2) this process.

The described correction step of using a short read assembly to identify and excise discordant portions of the long read assembly is arguably more aggressive compared with the use of programs such as GAM-NGS (Vicedomini *et al.* 2013), where different assemblies are merged to produce what is hoped to be a more accurate composite product. By using the short read assembly aggressively to remove potential misassembled regions, we erred on the side of accuracy at the expense of contig length. Moreover, the use of an assembly-merging program might obscure the study’s principal aim, which was to determine the standalone performance metrics for the direct assembly of low-coverage, noisy long reads.

### Assembly quality assessment and genome annotation

**Figure 6:**
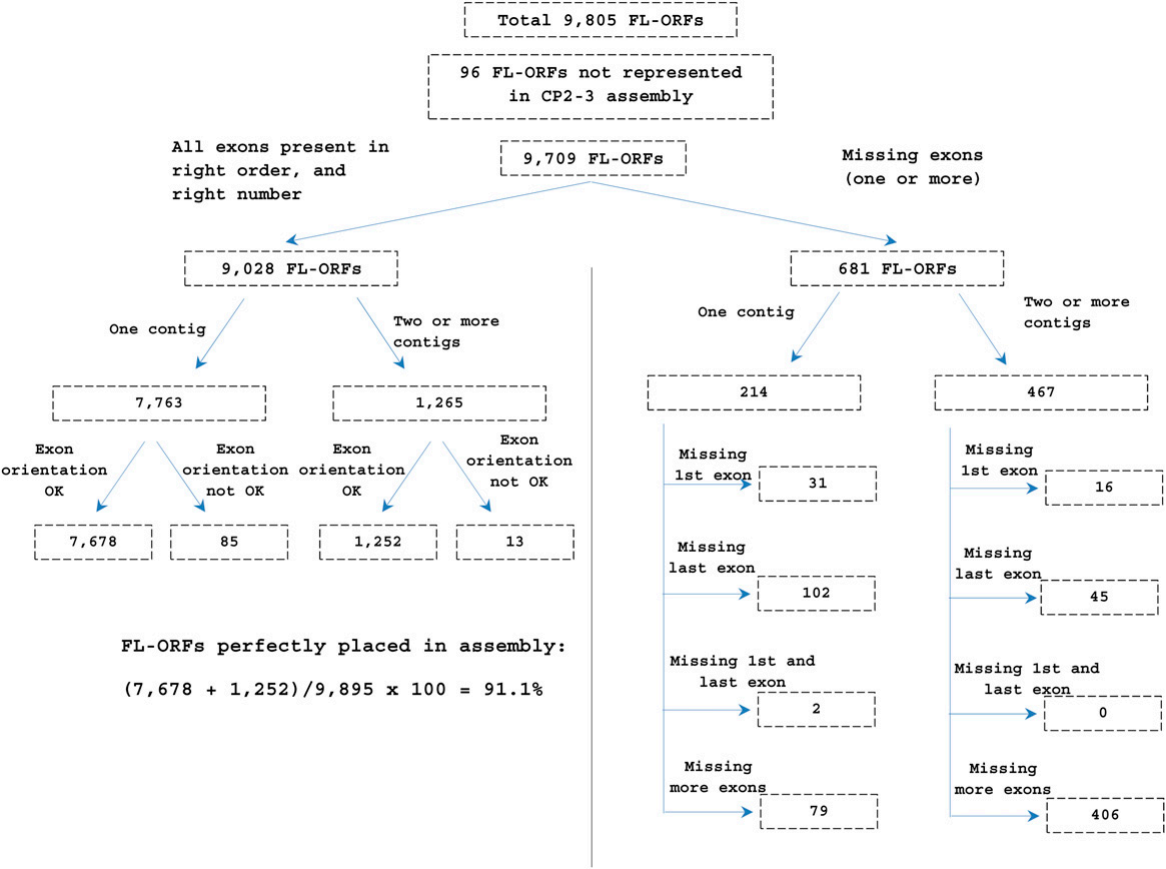
Reconciliation of genome scaffolded assembly CP2-3 with the exon–gene models of 9805 FL-ORFs. The exon–gene models of 8930 FL-ORFs (91.1%) were congruent with the CP2-3 assembly. The exons of 96 FL-ORFs (1%) were not found in the assembly. The remaining 779 FL-ORFs (7.9%) were missing one or more exons or have one or more exons in an incorrect orientation. FL-ORF, full-length open reading frame.

We used the constructed beaver transcriptome to gauge the completeness and accuracy of our genome assembly, and to annotate the assembled genome; 8534 (87%) of our reconstructed set of 9805 beaver FL-ORFs could be mapped precisely onto the CP2-2 assembly in complete concordance with the exon–gene model. This finding showed the good quality and the general completeness of the assembly at the level of individual exons, and at a larger scale, by the correct number, order, and orientation of the entire exon complement of each FL-ORF. The complete exon complement for 7759 FL-ORFs (79.1%) could be mapped onto single contigs in the CP2-2 assembly in concordance with the predicted exon–gene model. The complete exon complements of an additional 775 FL-ORFs (7.9%) could be similarly mapped, but the mapped exons spread over two or more contigs, thereby providing useful scaffolding information. However, our assembly was not perfect with respect to all our FL-ORFs: no exons for 353 FL-ORFs (3.6%) could be found in the CP2-2 assembly, and an additional 918 FL-ORFs (9.4%) have one or more exons missing or misorientated. Plausible explanations for the missing exons in CP2-2 might include insufficient sampling of the genome due to low sequence depth, misassembly of either the genome or the transcriptome, or the use of an incorrect exon–gene model. We looked at the secondary assemblies (CP Unused and AB2) for the missing exons. In this process, we achieved the correct and complete exon placement for 558 additional FL-ORFs from our collection. Contigs or DNA segments in the secondary assemblies bearing these exons were incorporated into the scaffold (see below), bringing the correct placement of FL-ORFs in our scaffolded assembly, CP2-3 (see below), to a total of 8930 (91.1%). Figure 6 summarizes the reconciliation of the beaver full-length transcriptome assembly with the scaffolded genome assembly, CP2-3.

**Figure 7:**
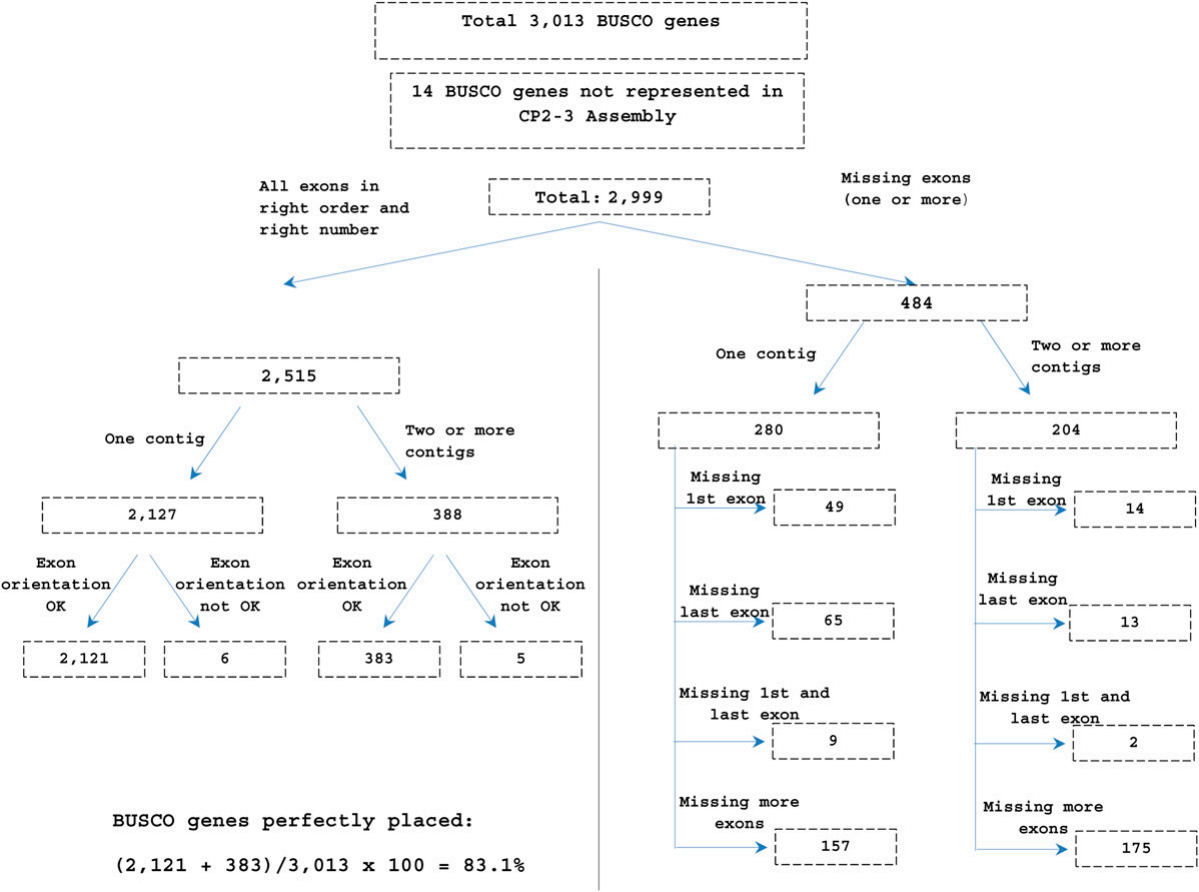
Reconciliation of genome assembly CP2-3 with the exon–gene models for each of the 3013 members of the BUSCO gene set. The exon–gene models of 2504 BUSCO genes (83.1%) were congruent with the CP2-3 assembly. The exons of 14 BUSCO genes (0.5%) were not found in the assembly. The remaining 495 BUSCO genes (16%) were missing one or more exons or had one or more exons in the incorrect orientation. BUSCO, Benchmarking Universal Single-Copy Orthologs.

In addition to our set of *de novo*-assembled beaver FL-ORFs, we used the BUSCO gene set (Simao *et al.* 2015) to gauge the completeness and accuracy of our CP2-2 assembly. The BUSCO gene set is a widely-used benchmark, comprising single-copy orthologous genes that are free of interference from paralogous sequences during mapping, and have a high degree of sequence conservation across multiple species. The mammalian BUSCO gene set comprises 3013 genes, of which 2113 were represented in our FL-ORF set derived from *de novo* assembly of the beaver leukocyte and muscle transcriptomes. Figure 7 summarizes the result of the full BUSCO gene set reconciled against the CP2-3 assembly. Of the 3013 BUSCO orthologs, nearly all (2999 entries, 99%) were represented in the CP2-3 assembly by at least one exon. The exon placements of 2504 BUSCO genes (83%) were complete and correct with respect to exon number, exon order, and orientation. The remaining 495 entries (17%) appeared as fragmented genes, with one or more exons missing, in the wrong order, or in an incorrect orientation (Figure 7). For the genes whose exon placements in our CP2-3 assembly were complete and correct, the results from the BUSCO set (83%) were slightly lower than from our full set of 9805 beaver FL-ORFs (91%). The reduced participation rate of the BUSCO set might stem from difficulties in cross-species detection of more divergent exons in our assembly, a problem that is not encountered for exons from our set of beaver FL-ORFs.

### Scaffolding of assembly

**Figure 8:**
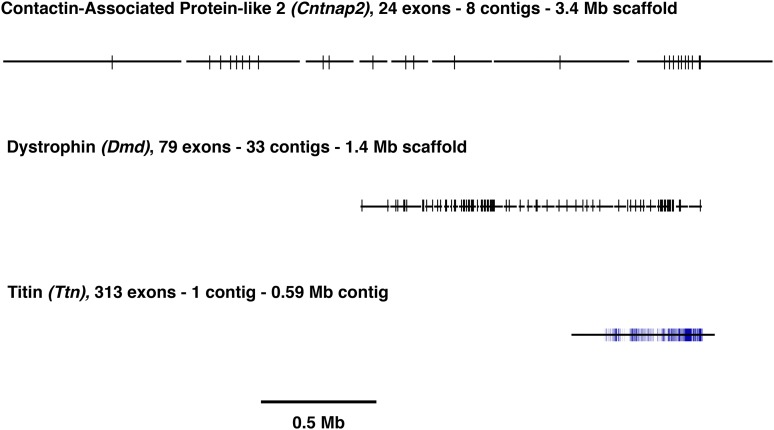
Diagrammatic depiction of *C. canadensis Cntnap2*, *Ddm*, and *Ttn* loci in assembly CP2-3. Vertical bars represent exons. Horizontal lines represent assembled contigs ordered in accordance with the exon–gene models for the indicated loci, providing scaffolding information.

The exon–gene models derived from our *de novo*-assembled set of 9805 FL-ORFs and 4045 PL-ORFs were used to produce the scaffolded assembly, referred to as “CP2-3.” The principle of the scaffolding process is illustrated using the full-length cDNA encoding dystrophin assembled from our beaver transcriptome data set (Table S1). Mutations in the dystrophin gene (*DMD*) cause a general class of myopathies, including Duchenne and Becker Muscular Dystrophy [review: Blake *et al.* (2001)]. The human *DMD* locus is one of the largest known, encoding a 3685 amino acid polypeptide translated from 79 exons spread over 2.2 Mb of the genome. Using the rodent-derived exon–gene model for *Dmd*, its 79 exons on 33 contigs in the CP2-2 assembly were consolidated into a single 1.4 Mb scaffold in the CP2-3 assembly. We repeated this process for *Cntnap2*, a member of the neurexin superfamily, and the largest known rodent gene (Nakabayashi and Scherer 2001). The 24 exons of the beaver *Cntnap2* locus were spread over eight contigs in our assembly, but could be similarly consolidated into a single scaffold of 3.4 Mb using the exon–gene model. We also examined the gene encoding Titin (Ttn), the largest known protein. The beaver *Ttn* locus of 313 coding exons was found on a single 590 kb contig in the CP2-2 assembly. While *Ttn* did not contribute to the scaffolding process, the complete assembly of this locus with its multiple repeating PEVK motifs, responsible for the protein’s elastic properties (Freiburg *et al.* 2000), reflected the accuracy and the utility of assembly using long reads. Figure 8 shows the schematics of the three loci in the CP2-3 assembly to illustrate the scaffolding process.

Table 6 summarizes the statistics of the scaffolding process as applied to the remainder of our assembled ORFs whose exon complement spanned multiple contigs. Figure 5 shows the size distribution of assembled contigs following scaffolding with the FL- and PL-ORFs. The scaffolded assembly, CP2-3, exhibited a reduction in contig number from 22,502 in CP2-2 to 21,157 in CP2-3, and a concomitant improvement in the N50 metrics from 278,680 to 317,558 bp (Table 6). This result was useful but relatively modest since only 1252 FL- and 84 PL-ORFs participated in the scaffolding process. The length profile of the scaffolded contigs is shown in Figure 5C. Similarly, scaffolding the Abyss assembly, AB2, using the transcriptome produced only a relatively marginal improvement in the assembly metrics in the short read assembly, from an N50 of 17,517 bp to an N50-scaffold of 19,662 bp (Table 6).

**Table 4:** Variants observed between Ward and Horn *et al.* (2011) beaver mitochondrial assemblies

Variant Start	Variant End	Length	Amino Acid Change	CDS Position	Change	Codon Change	Polymorphism Type
1,680	1,680	1			C→T		SNP (transition)
2,209	2,208	0			-TTC		Deletion
2,630	2,630	1			A→T		SNP (transversion)
2,786	2,786	1		30	C→T	ACC→ACT	SNP (transition)
3,200	3,200	1		444	C→T	ATC→ATT	SNP (transition)
3,296	3,296	1		540	G→A	CCG→CCA	SNP (transition)
3,323	3,323	1		567	A→G	ACA→ACG	SNP (transition)
3,668	3,668	1		912	T→C	TAT→TAC	SNP (transition)
3,688	3,688	1	T→M	932	C→T	ACA→ATA	SNP (transition)
4,605	4,605	1		684	T→C	CTT→CTC	SNP (transition)
4,674	4,674	1		753	C→T	CTC→CTT	SNP (transition)
4,770	4,770	1		849	C→T	GCC→GCT	SNP (transition)
5,178	5,178	1			T→C		SNP (transition)
5,409	5,409	1		66	T→C	TTT→TTC	SNP (transition)
5,541	5,541	1		198	T→C	ATT→ATC	SNP (transition)
5,652	5,652	1		309	A→G	TGA→TGG	SNP (transition)
6,312	6,312	1		969	G→A	TGG→TGA	SNP (transition)
6,360	6,360	1		1017	C→T	CTC→CTT	SNP (transition)
6,522	6,522	1		1179	T→C	TTT→TTC	SNP (transition)
7,043	7,043	1		13	C→T	CTA→TTA	SNP (transition)
7,453	7,453	1		423	A→G	CGA→CGG	SNP (transition)
7,489	7,489	1		459	A→G	CTA→CTG	SNP (transition)
7,834	7,834	1		51	T→C	ATT→ATC	SNP (transition)
7,909	7,909	1		126	T→C	GTT→GTC	SNP (transition)
8,013	8,013	1		69	T→C	ATT→ATC	SNP (transition)
8,284	8,284	1	I→V	340	A→G	ATT→GTT	SNP (transition)
8,651	8,651	1		27	T→C	CAT→CAC	SNP (transition)
8,702	8,702	1		78	C→T	CTC→CTT	SNP (transition)
8,942	8,942	1		318	G→A	CTG→CTA	SNP (transition)
9,356	9,356	1		732	C→T	TTC→TTT	SNP (transition)
10,013	10,013	1		120	T→C	ATT→ATC	SNP (transition)
10,019	10,019	1		126	T→C	ATT→ATC	SNP (transition)
10,036	10,036	1	V→A	143	T→C	GTC→GCC	SNP (transition)
10,082	10,082	1		189	T→C	ATT→ATC	SNP (transition)
10,112	10,112	1		219	A→G	GTA→GTG	SNP (transition)
10,519	10,519	1		336	C→T	GCC→GCT	SNP (transition)
10,591	10,591	1		408	A→G	TGA→TGG	SNP (transition)
10,690	10,690	1	N→K	507	C→A	AAC→AAA	SNP (transversion)
11,528	11,528	1		1345	T→C	TTA→CTA	SNP (transition)
11,734	11,734	1			(A)5→(A)6		Insertion (tandem repeat)
12,517	12,517	1		759	A→G	GTA→GTG	SNP (transition)
13,366	13,366	1		1608	G→A	TCG→TCA	SNP (transition)
13,945	13,945	1			G→A		SNP (transition)
14,005	14,005	1			A→G		SNP (transition)
14,466	14,466	1	I→V	304	A→G	ATC→GTC	SNP (transition)
14,637	14,637	1	D→N	475	G→A	GAC→AAC	SNP (transition)
14,703	14,703	1	F→L	541	T→C	TTC→CTC	SNP (transition)
14,837	14,837	1		675	C→T	ACC→ACT	SNP (transition)
14,954	14,954	1		792	T→C	ACT→ACC	SNP (transition)
15,356	15,356	1			(A)5→(A)6		Insertion (tandem repeat)
15,515	15,515	1			T→C		SNP (transition)
15,544	15,544	1			T→C		SNP (transition)
15,632	15,632	1			T→A		SNP (transversion)
15,634	15,634	1			G→A		SNP (transition)
15,674	15,674	1			T→C		SNP (transition)
15,685	15,685	1			G→A		SNP (transition)
15,692	15,692	1			C→T		SNP (transition)
15,755	15,755	1			C→T		SNP (transition)
15,766	15,766	1			C→T		SNP (transition)
15,813	15,813	1			G→A		SNP (transition)
15,815	15,815	1			A→G		SNP (transition)
15,817	> 15817	> 1			GT→A		Deletion
15,907	15,907	1			+A		Insertion
16,376	16,439	64			(ACACGTATACACGTATACACGTATACACGTAT)8→(ACACGTATACACGTATACACGTATACACGTAT)10		Insertion (tandem repeat)
16,504	16,504	1			A→C		SNP (transversion)
16,507	16,509	3			+ACC		Insertion
16,527	16,528	2			GC→CG		Substitution
16,689	16,689	1			A→G		SNP (transition)

CDS, coding sequence; SNP, single nucleotide polymorphism.

**Table 5:** Variants observed between Ward and ROM beaver mitochondrial assemblies

Variant Start	Variant End	Length	Amino Acid Change	CDS Position	Change	Codon Change	Polymorphism Type
1,033	1,033	1			A→G		SNP (transition)
3,107	3,107	1		351	G→A	CTG→CTA	SNP (transition)
3,296	3,296	1		540	A→G	CCA→CCG	SNP (transition)
3,347	3,347	1		591	A→C	CCA→CCC	SNP (transversion)
4,047	4,047	1		126	C→A	CCC→CCA	SNP (transversion)
4,770	4,770	1		849	T→C	GCT→GCC	SNP (transition)
4,810	4,810	1	V→I	889	G→A	GTC→ATC	SNP (transition)
5,541	5,541	1		198	C→T	ATC→ATT	SNP (transition)
6,522	6,522	1		1179	C→T	TTC→TTT	SNP (transition)
6,804	6,804	1		1461	T→C	CTT→CTC	SNP (transition)
7,043	7,043	1		13	T→C	TTA→CTA	SNP (transition)
7,834	7,834	1		51	C→T	ATC→ATT	SNP (transition)
7,909	7,909	1		126	C→T	GTC→GTT	SNP (transition)
8,284	8,284	1	V→I	340	G→A	GTT→ATT	SNP (transition)
8,702	8,702	1		78	T→C	CTT→CTC	SNP (transition)
9,959	9,959	1		66	T→C	TAT→TAC	SNP (transition)
10,013	10,013	1		120	C→T	ATC→ATT	SNP (transition)
10,019	10,019	1		126	C→T	ATC→ATT	SNP (transition)
10,112	10,112	1		219	G→A	GTG→GTA	SNP (transition)
10,288	10,288	1		105	T→C	AGT→AGC	SNP (transition)
11,528	11,528	1		1345	C→T	CTA→TTA	SNP (transition)
12,517	12,517	1		759	G→A	GTG→GTA	SNP (transition)
13,764	13,764	1	N→Y	325	T→A	AAT→TAT	SNP (transversion)
14,687	14,687	1		525	A→G	CTA→CTG	SNP (transition)
14,703	14,703	1	L→F	541	C→T	CTC→TTC	SNP (transition)
14,837	14,837	1		675	T→C	ACT→ACC	SNP (transition)
14,954	14,954	1		792	C→T	ACC→ACT	SNP (transition)
15,544	15,544	1			C→T		SNP (transition)
15,561	15,561	1			T→C		SNP (transition)
15,618	15,618	1			C→T		SNP (transition)
15,634	15,634	1			A→G		SNP (transition)
15,692	15,692	1			T→C		SNP (transition)
15,702	15,702	1			A→G		SNP (transition)
16,136	16,136	1			T→C		SNP (transition)

CDS, coding sequence; SNP, single nucleotide polymorphism.

**Table 6:** Statistics for *C. canadensis* genome scaffolded assemblies CP2-3 and AB2-3 using FL- and PL-ORFs

	Assembly	Scaffolded Assembly
PacBio-Canu assembly CP2-3		
Number of contigs/scaffolds	22,502	21,157
Longest contig/scaffold	3,330,706 bp	4,235,261 bp
Span (total bp)	2.486 Gb	2.515 Gb
Mean contig/scaffold length	110,503 bp	119,017 bp
N50 contig/N50 scaffold	278,680 bp	317,558 bp
Abyss assembly AB2-3		
Number of contigs/scaffolds	316,144	300,561
Longest contig/scaffold	206,011 bp	787,257 bp
Span (total bp)	2.431 Gb	2.432 Gb
Mean contig/scaffold length	7,688 bp	8,091 bp
N50 contig/N50 scaffold	17,517 bp	19,662 bp

### Genome annotation

We identified known repeat elements described in RepBase in CP2-3 using RepeatMasker (Tarailo-Graovac and Chen 2009). Table 7 summarizes the repeat distributions in the beaver draft genome. Matches covered 30.4% of the genome, including 6.6% SINEs, 15.1% LINEs, 4.9% LTR elements, and 1.25% simple repeats, with the remainder being of other repeat classes. BLASTn analysis of the Ward mitochondrial genome (mito-1) against the CP2-3 assembly revealed 193 potential mitochondrial pseudogene loci (data not shown). These so-termed “NUMTs” are believed to originate from invasion of the nuclear genome by mitochondrial DNA via nonhomologous recombination [for a review, see Richly and Leister (2004)]. It is generally viewed that the accumulation of NUMTs is a continuous evolutionary process (Triant and DeWoody 2007). Using the same BLASTn threshold of 1e-5, the mouse (*M. musculus*) has 190 copies and the rat (*R. norvegicus*) has 61 copies (Richly and Lesiter 2004).

**Table 7:** RepeatMasker results for *C. canadensis* assembly CP2-3

	Number of Elements*^a^*	Length Occupied (bp)	Percentage of Sequence (%)
SINEs	1,526,194	167,113,901	6.64
Alu/B1	320,643	31,356,476	1.25
B2-B4	124,373	7,917,276	0.31
IDs	441,613	37,889,991	1.50
MIRs	232,410	32,110,048	1.28
LINEs	584,641	379,985,492	15.09
LINE1	420,271	340,564,285	13.52
LINE2	143,179	35,333,421	1.40
L3/CR1	16,864	3,272,497	0.13
LTR elements	358,396	123,574,660	4.91
ERVL	72,861	26,961,457	1.07
ERVL-MaLRs	200,863	68,820,344	2.73
ERV_classI	43,815	20,130,270	0.80
ERV_classII	28,700	4,316,563	0.17
DNA elements	221,280	48,541,417	1.93
hAT-Charlie	123,347	24,927,384	0.99
TcMar-Tigger	45,279	12,145,600	0.48
Unclassified	6,070	1,176,346	0.05
Total interspersed repeats		720,391,816	28.61
Small RNA	59,721	4,937,299	0.20
Satellites	5,814	820,000	0.03
Simple repeats	655,839	31,533,733	1.25
Low complexity	159,452	9,064,948	0.36

The query species was assumed to be rodentia. RepeatMasker version open-4.0.5, rushjob mode run with rmblastn version 2.2.27+, RepBase Update 20140131, RM database version 20140131. File name: cp3.scaff.assembly.fasta; sequences, 21,170; total length, 2,518,060,007 bp (2,517,076,473 bp excluding N/X-runs); GC level, 39.72%; and bases masked, 765,530,353 bp (30.40%).

a Most repeats fragmented by insertions or deletions have been counted as one element.

We also annotated our assembled beaver genome with respect to loci involved in dentition. Rodents are taxonomically distinguished by a characteristic pair of continuously growing sharp incisors. Rodent dentition is highly regulated (for reviews see: Bei 2009; Caton and Tucker 2009; Peters and Balling 1999; Rodrigues *et al.* 2013). In particular, the beaver incisors are well-developed and have highly specialized enamel, reflecting their continuous need to fell trees. Relevant to beaver dentition is that enamel deposition occurs only on the front (labial) surface, resulting in a continuously sharp tree cutting edge as the softer inner (lingual) surface of the incisor is differentially worn by gnawing. Selective enamel deposition in rodent teeth is due to asymmetrical expression of follistatin (*Fst*), an antagonist of BMP signaling and ameloblast differentiation, at the lingual dental epithelium (Wang *et al.* 2004). Rodent enamel is also often pigmented due to small amounts of substituting ions, such as iron, in the hydroxylapatite lattice, thereby increasing acid resistance and mechanical strength. High iron levels give beaver incisors their characteristic orange hue (Gordon *et al.* 2015). Iron is derived from blood plasma, and the iron carrier protein, Fth, is encoded by one of the most highly upregulated genes in rodent ameloblasts (Wen and Paine 2013). Two transcription factors appear to be critical for enamel deposition; *Tbx1*-deficient mice produce no enamel (Caton *et al.* 2009) and the enamel of *Nrf2*-deficient mice fails to incorporate iron (Yanagawa *et al.* 2004). To provide a genomics resource to study dentition, Table 8 presents the GenBank accession numbers for the complete coding sequence and exon structure for *Fst*, *Bmp4*, *Bmp7*, *Fth*, *Tbx1*, and *Nrf2* (also known as *Nef2l2*) loci described above, and those of 34 other beaver loci present in the CP2-3 assembly whose orthologs have been shown in rodent models to be important in different stages of dentition.

**Table 8:** Beaver loci involved in dentition

Gene	Gene Name	Reference	Accession
Enzyme
* Klk4*	Enamel Matrix Protease	Lu *et al.* (2008)	KY286078
* Mmp14*	Matrix Metallopeptidase 14	Yoshiba *et al.* (2003)	KY286080
* Mmp20*	Enamelysin/Matrix Metallopeptidase 20	Lu *et al.* (2008)	KY286081
Extracellular Matrix Protein
* Ambn*	Ameloblastin	Fukumoto *et al.* (2004)	KY286056
* Amelx*	Amelogenin X Chromosome	Sasaki and Shimokawa (1995)	KY286057
* Amtn*	Amelotin	Abbarin *et al.* (2014)	KY286058
* Dmp1*	Dentin Matrix Protein 1	Gibson *et al.* (2013)	KY286066
* Dspp*	Dentin Sialophosphoprotein	Gibson *et al.* (2013)	KY286067
* Enam*	Enamelin	Masuya *et al.* (2005)	KY286070
Signal Transduction
* Bmp4*	Bone Morphogenic Protein 4	Plikus *et al.* (2005)	KY286062
* Bmp7*	Bone Morphogenic Protein 7	Plikus *et al.* (2005)	KY286063
* Dkk1*	Dickkopf-Related Protein 1	Fjeld *et al.* (2005)	KY286065
* Eda*	Ectodysplasin A	Tucker *et al.* (2000)	KY286068
* Edar*	Ectodysplasin A Receptor	Tucker *et al.* (2000)	KY286069
* Fst*	Follistatin	Wang *et al.* (2004)	KY286071
* Grem2*	Gremlin 2	Vogel *et al.* (2015)	KY286073
* Inhba*	Inhibin A	Ferguson *et al.* (1998)	KY286077
* Nog*	Noggin	Hu *et al.* (2012)	KY286086
* Wnt10a*	Int/Wingless 10a	Yamashiro *et al.* (2007)	KY286096
* Wnt6*	Int/Wingless 6	Yamashiro *et al.* (2007)	KY286095
Structural Protein
* Ibsp*	Integrin Binding Sialoprotein	MacNeil *et al.* (1995)	KY286076
* Tfip11*	Tuftelin-Interacting Protein 11	Paine *et al.* (2000)	KY286092
* Tuft1*	Tuftelin	Zeichner-David *et al.* (1997)	KY286094
Transcription Factor
* Barx1*	BarH-like Homeobox 1	Tucker *et al.* (1998)	KY286060
* Bmi1*	Polycomb Complex Protein BMi1	Biehs *et al.* (2013)	KY286061
* Hand1*	Heart and Neural Crest Derivatives 1	Abe *et al.* (2002)	KY286074
* Hand2*	Heart and Neural Crest Derivatives 2	Abe *et al.* (2002)	KY286075
* Lef1*	Lymphoid Enhancer-Binding Factor 1	Sasaki *et al.* (2005)	KY286079
* Msx1*	Muscle Segment Homeobox 1	Bei *et al.* (1998)	KY286082
* Msx2*	Muscle Segment Homeobox 2	Aioub *et al.* (2007)	KY286083
* Nfic*	Nuclear Factor 1 C-Type	Kim *et al.* (2015)	KY286085
* Nfe2L2*	Nuclear Factor (Erythroid-Derived 2)-like 2	Yanagawa *et al.* (2004)	KY286084
* Pax6*	Paired Box Protein Pax 6	Kaufman *et al.* (1995)	KY286087
* Pax9*	Paired Box Protein Pax 9	Peters *et al.* (1998)	KY286088
* Pitx1*	Paired-like Homeodomain Transcription Factor 1	Mitsiadis and Drouin (2008)	KY286089
* Pitx2*	Paired-like Homeodomain Transcription Factor 2	Liu *et al.* (2003)	KY286090
* Tbx1*	T-Box Transcription Factor TBX1	Caton *et al.* (1999)	KY286091
Other
* Atp6V0a1*	ATPase, H+ Transporting, lysosomal V0 subunit A1	Sarkar *et al.* (2016)	KY286059
* Fth1*	Ferritin Heavy Chain	Wen and Paine (2013)	KY286072
* Timp2*	Tissue Inhibitor of Metallopeptidase 2	Yoshiba *et al.* (2003)	KY286093

**Figure 9:**
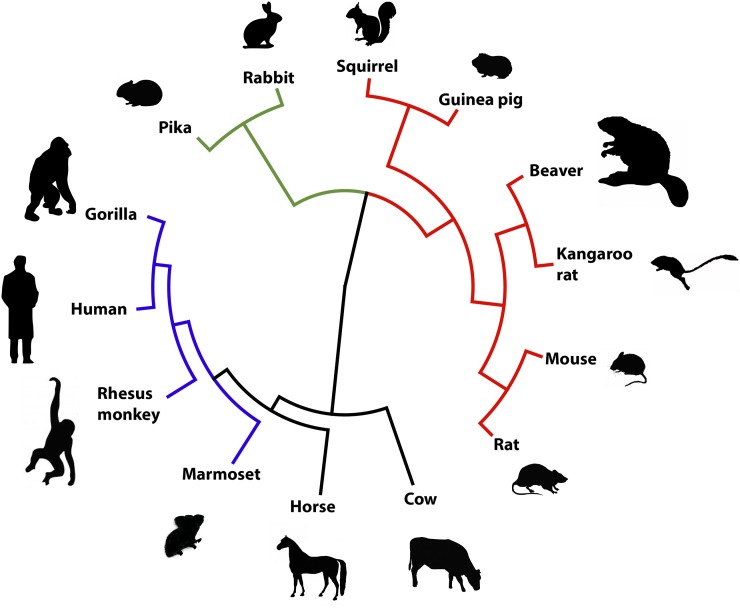
Tree based on 507 amino acid alignment of *C. canadensis* Dmp-1 to 13 other mammalian Dmp1 protein sequences: XP_012885281 Ord’s kangaroo rat (*D. ordii*); NP_058059 house mouse (*M. musculus*); XP_008768217.1 Norway brown rat (*R. norvegicus*); XP_017202907.1 European rabbit (*Oryctolagus cuniculus*); XP_004590692.1 American pika (*Ochotona princeps*); XP_005341957.1 13 lined ground squirrel (*I. tridecemlineatus*); XP_003469412.1 guinea pig (*Cavia porcellus*); NP_004398.1 man (*H. sapiens*); XP_004039140.1 Western lowland gorilla (*Gorilla gorilla gorilla*); XP_014994254.1 rhesus macaque (*Macaca mulatta*); XP_002745661.1 common marmoset (*Callithrix jacchus*); XP_005608699.1 horse (*Equus caballus*); and NP_776463.2 domestic cow (*Bos taurus*). Tree was constructed using PhyML 3.0 (Guindon *et al.* 2010) with the JTT model of amino acid substitution. Color legend: red = Rodentia; blue = Primate; green = Lagomorpha.

As a preliminary glance at how our beaver sequences might contribute to the evolutionary history of dentition, we examined one representative gene, *Dmp1*. Dmp1 (dentin matrix acidic phosphoprotein 1) is an extracellular protein involved in mineralization of dentin and is also a regulator of *Dspp* (dentin sialophosphoprotein), both of which are critical for rodent teeth formation (Gibson *et al.* 2013). We aligned the 507 amino acid sequence encoded by the predicted *C. canadensis Dmp1* gene to 13 other mammalian Dmp1 proteins downloaded from GenBank. Using the program PhyML 3.0 (Guindon *et al.* 2010) with the JTT model of amino acid substitution, we constructed a tree for Dmp1 (Figure 9). The branch tips of the Dmp1 tree agree with the recent eutherian mammalian phylogeny based on 477 nuclear genes (Song *et al.* 2012). Our data specifically placed beaver Dmp1 in proximity with Dmp1 of Ord’s kangaroo rat (*D. ordii*), for which partial genomic sequence is available (www.ncbi.nih.gov/genome/772). Together with the kangaroo rat, the present study provides important genomic resources to study this region of the rodent phylogenetic tree.

### Residual PacBio sequencing errors in the CP2-3 assembly

Raw PacBio reads are generally reported to have a 15–20% error rate (Chaisson and Tesler 2012; Koren *et al.* 2012). The error profile for raw PacBio reads has been extensively modeled (Ono *et al.* 2013). Unlike other sequencing platforms, PacBio errors appeared to be more or less randomly distributed along the entire length of the read, typically comprising ∼11% insertions, 4% deletions, and 1% substitutions. We made use of our annotated gene set in Table 8 to estimate the residual PacBio sequence errors remaining in our assembly that survived the Canu assembly and the subsequent polishing steps. We compared the coding sequences for these genes from the CP2-3 assembly with the consensus sequence derived from Illumina reads (80-fold coverage) tiled over the same regions of CP2-3. The combined coding exons of the 40 loci in Table 8, including the addition of *Cntnap2*, covered 46,354 bp on the CP2-3 assembly. Benchmarked against the Illumina consensus sequence, we observed only seven single-base indels, and one two-base insertion. We observed no base substitutions indicative of residual sequencing errors in the CP2-3 assembly when compared with the Illumina consensus sequence after accounting for the allelic substitutions. Together, the identified indels and the absence of base-substituted positions were consistent with a residual sequence error rate of < 0.02% in the region investigated. This result indicated that two rounds of polishing using Pilon supported by short Illumina reads were effective in removing nearly all PacBio sequencing errors.

### Conclusions

Assembly from long reads has notable advantages over short read assembly in terms of repeat resolution and phasing, to name just a few. However, long read technologies are currently limited by cost, throughput, and accuracy. We showed the feasibility of assembling a complex, mammalian genome directly from moderate-coverage of long noisy reads, to produce a useful draft assembly. We expect that further development of this approach will allow it to be scaled to large projects, facilitated by the continued cost reduction and performance improvements in long read sequencing.

Our beaver sequence assemblies provide a high-resolution genomic framework, complementing earlier karyotyping (Lavrov and Orlov 1973; Genest *et al.* 1979) for the study of rodent evolution. Genomic cross-talk between the host nuclear, mitochondrial, and the microbial genomes is now widely studied, and along with environment factors, are recognized for driving adaptive evolution (reviewed in Levy *et al.* 2015). As such, our report of the beaver genome complements recent metagenomic studies of the gastrointestinal microbiome in this species in response to its lignocellulose-rich diet (Gruninger *et al.* 2016; Wong *et al.* 2016).

In addition to genomic cross-talk, genomes may also coevolve, a concept underlying the emerging field of “cultural genomics” (Diamond 1997; see reviews, Wade 2007; Laland *et al.* 2010; Foote *et al.* 2016). The close economic and cultural interactions of beaver and man over the past several hundred or more years may be contributing to the shaping of the genomes of both species. Aggressive economic pursuit and conflict over beaver pelts has most certainly contributed to human gene flow throughout North America. Similarly, beaver subspecies that might have previously existed are now either extinct by over-trapping driven by European fashion trends, or by habitat loss from human encroachment. Moreover, other beaver gene pools were supplanted by the deliberate introduction of the Canadian beaver halotype (*C. canadensis canadensis*) from the Hudson’s Bay region, which has the most prized fur (Carlos and Lewis 2011). This complex interplay of environmental, metagenomic, cultural/economic, and other still-to-be determined influences on genomic selection and animal and human behavior, has had a profound influence on Canadian and world history. Our draft *de novo* assembly of the beaver genome and accompanying transcriptome data could provide new insights into this evolutionary interplay.

## Supplementary Material

Supplemental material is available online at www.g3journal.org/lookup/suppl/doi:10.1534/g3.116.038208/-/DC1.
